# Enhancing the Participation of African Americans in Health-Related Genetic Research: Findings of a Collaborative Academic and Community-Based Research Study

**DOI:** 10.1155/2013/749563

**Published:** 2013-12-04

**Authors:** Sandra Millon Underwood, Aaron G. Buseh, Sheryl T. Kelber, Patricia E. Stevens, Leolia Townsend

**Affiliations:** ^1^University of Wisconsin-Milwaukee College of Nursing, 1921 E Hartford Avenue, P.O. Box 413, Cunningham Hall, Room 422/423, Milwaukee, WI 53201, USA; ^2^University of Wisconsin-Milwaukee College of Nursing, Center for Nursing Research and Evaluation, WI 53201, USA

## Abstract

The involvement of African Americans in research has long been expressed as a concern by the scientific community. While efforts have been undertaken to identify factors inhibiting the participation of African Americans in health-related research, few efforts have been undertaken to have highlight factors associated with their engagement of health-related research. An exploratory study of factors presumed to be associated with participation in health-related research was conducted among a nonprobability sample of African Americans (*n* = 212) from a large urban community in the Midwest. The study was guided by a framework that hypothesized the influence of knowledge, beliefs, and perceptions about genetics and the involvement of providers in decision-making on willingness to participate in health-related genetic research. The results revealed that knowledge, beliefs, and perceptions about genetics and the involvement of providers were associated with willingness to engage in health-related genetic research (*P* < .05). The most interesting, however, was that 88.7% of the participants who had not previously been involved in a health-related study who expressed a willingness to participate reported that they “had never been asked.” Study findings suggest the need for research that further examines factors associated with the involvement of African Americans in health-related genetic research.

## 1. Introduction

**Figure 1:**
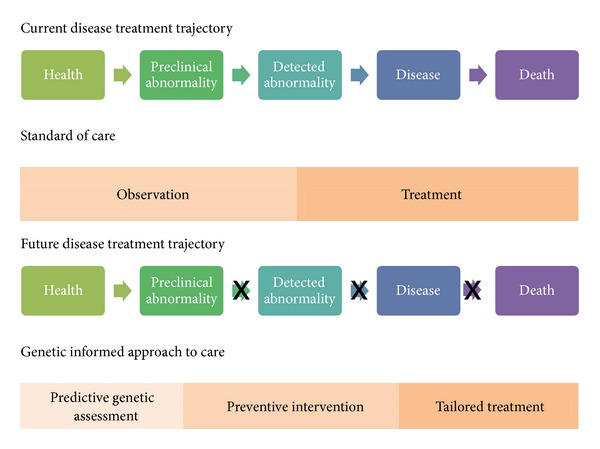
Disease treatment trajectory.

In April 2003 the directors of the Human Genome Project (HGP), an international scientific research project coordinated by the United States Department of Energy and the National Institutes of Health National Human Genome Research Institute, announced that the first draft of the map of the human genome had been completed [1]. It was anticipated that mapping the complete set of DNA would revolutionize health care and lay the groundwork for the development of clinical markers with predictive capabilities and, thereby, shift the disease-treatment trajectory and lead to preventive interventions, tailored treatments, and averted deaths (Figure 1). Likewise, it was anticipated that the map would lead to a better understanding of the causes of cardiac disease, cancer, diabetes, Alzheimer's disease, mental disorders, and other common and rare diseases; the development of diagnostic tests to detect errant genes; the development of new classes of medicines based on gene sequence and protein structure function; and the development of therapies which use genes in treating genetic and acquired diseases [2, 3].

For population groups known to experience excess disease-related morbidity, and mortality it was believed that the ability to use genetics to predict, prevent, detect, and more effectively treat disease held tremendous promise. Ten years after completion of the map, HGP leaders report several accomplishments of the HGP. They report the identification of approximately 1,800 disease genes [4, 5], the development of more than 2,000 genetic tests for various human diseases/conditions [6], and the development and ongoing testing of more than 350 biotechnology-based genetic products [4]. Yet, in a more subdued voice, they note that the anticipated clinical benefit from the HGP has yet to unfold [3].

Several public and private entities across the country have been established and have begun collecting and storing tissue specimens for genetic testing to support and expand the program of study initiated by the HGP. According to reports authored by respected scientists in the field of genetics, in 1999 close to 300 million tissue samples—most of which were collected during routine clinical and surgical procedures—were stored in public health departments, blood banks, pathology archives, and researchers' laboratories within the United States [7–10]. It has been estimated that the number of tissue samples collected since that time has increased by more than 20 million a year [11].

Many in the scientific, medical, and advocacy arena, concerned about the excess disease-related morbidity and mortality and health disparities experienced by African Americans and other racial/ethnic minority populations, believe that the inclusion of biological specimens (and other health-related data) from African American and other racial/ethnic minority populations is essential [12–14]. However, an ever increasing number of reports allude to the limited inclusion of biological specimens (and other health-related data) from ethnic/racial minorities in biorepositories [15–23].

## 2. Purpose 

The manner and degree to which African Americans have been involved in medical research—a population often cited as being unduly burdened by disease—have long been expressed as a concern by leaders in the scientific community. While several efforts have been undertaken to identify factors inhibiting the participation of African Americans in health-related research, few efforts have been undertaken to have highlight factors associated with the engagement of African Americans in health-related research and gene testing [15, 22–30]. A focused study of factors presumed to be associated with the participation of African Americans in health-related genetic research was therefore proposed. The study was conducted by a team of nurse scientists, health educators, clinicians, and biostatisticians using principles of community engagement and community-based research [31, 32]. The study was designed to assess factors associated with the engagement of African Americans in health-related research among a targeted group of African-American men and women. More specifically, the study was designed to assess the influence of knowledge about genetics; beliefs regarding benefits and risks of gene testing; perceptions regarding the utility of genetic testing; and the involvement of health care providers in decisions regarding participation in health-related genetic research and their willingness to participate in health-related research that involved gene analysis.

**Figure 2:**
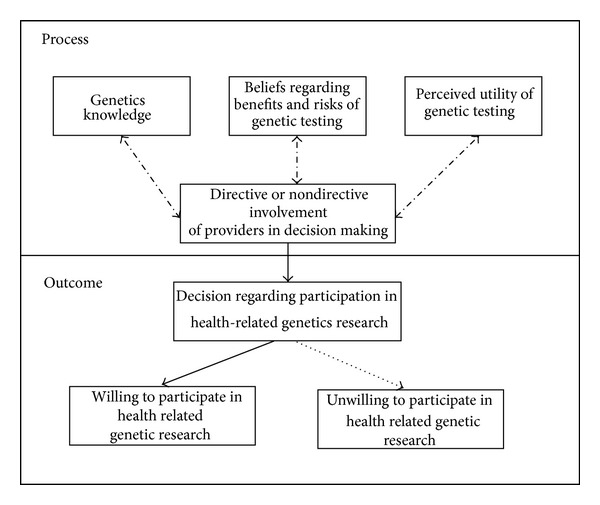
Organizing framework.

The organizing framework designed for the study included constructs deemed to be essential to informed decision-making and engagement in health-related genetic research [4, 33–36] (Figure 2). In applying the framework in this study, the independent process variables were knowledge about genetics; beliefs regarding the benefits and risks of gene testing; perceptions regarding the utility of genetic testing; and involvement of health care providers in health care decisions. The dependent outcome variable was willingness to participate in health-related genetic research. More specifically, the organizing framework hypothesized the influence of knowledge about genetics; beliefs regarding benefits and risks of gene testing; perceptions regarding the utility of genetic testing; and directive and nondirective involvement of health care providers on decisions made relative to participation in health-related genetics research and willingness to participate in health-related genetic research.

## 3. Methods 

### 3.1. Design

An exploratory cross-sectional study design was used to examine the influence of knowledge about genetics; beliefs regarding benefits and risks of gene testing; perceptions regarding the utility of genetic testing; and the involvement of health care providers on decisions made relative to participation in health-related genetics research and willingness to participate in health-related genetic research among a targeted group of African American men and women.

### 3.2. Sample, Setting, and Data Collection

A nonprobability sample of African American men and women who resided in a large, densely populated, economically, socially, and culturally diverse urban community in the Midwest was recruited to the study. Prospective participants who were 18 years of age or older; able to communicate in English; willing to complete a questionnaire about gene testing and genetic research; and able and willing to consent to participate in the study were invited to participate in the study by members of the research team. The investigative team worked in collaboration with the Black Health Coalition of Wisconsin (BHCW) to recruit study participants. Four community leaders, identified by the coalition director, were hired to and served as facilitators and recruiters for the study. After completing an online module on the protection of human subjects, each study facilitator was provided an overview of the study protocol and an overview of the procedures and methods used for data collection. Flyers and announcements explaining the project and providing contact information were posted by the study facilitators at community centers, heath centers, social service centers, and other public venues frequented by diverse groups of African American men and women.

Individuals expressing an interest in participating in the study were contacted by a study facilitator. After which, a meeting was arranged to further discuss the purpose of the study, to describe the procedures to be used in gathering study data, and to obtain their written consent to participate in the study. To facilitate the collection of the study data, the questionnaire was administered by the study facilitators. Prospective participants were informed that completion of the study questionnaire was voluntary and that receipt of services and/or support at the recruitment sites was not contingent on their participation. Prospective participants were informed that no names or personal identifiers would be requested or recorded. Data were collected between June 2010 and January 2012.

### 3.3. Measures

An investigator-designed questionnaire was developed to collect the study data. Included in the questionnaire were quantitative measures relevant to involvement of providers in health care decisions and decisions made by regarding participation in health related genetic research. Quantitative measures developed, validated and used in national cohort studies to assess genetics knowledge, beliefs regarding the merits and risks of genetic testing, and perceptions regarding the utility of genetic testing [37–42] were also included.

*(i) Knowledge about Genetics.* Seventeen items were used to assess knowledge about genetics. Eleven true-false items were used to assess knowledge of genetics principles and implications; two items were used in which participants were asked to describe how much they felt they knew about genetics; and four forced-choice items were used to assess knowledge of ongoing health-related genetic studies being conducted within the region.

*(ii) Beliefs Regarding the Benefits and Risks of Gene Testing*. Nineteen items were used to assess benefits and risks of gene testing. The items included statements suggesting the benefits, anticipated consequences, and negative impact of gene testing in which participants were asked to describe their beliefs using a 5-point Likert scale (1 = strongly disagree to 5 = strongly agree).

*(iii) Perceived Utility of Gene Testing*. Six items were used to assess perceptions regarding the utility of gene testing. Items inferring the importance of gene testing for risk assessment, screening, early detection, and treatment were included.

*(iv) Involvement of Health-Care Providers in Health Care Decisions*. Twenty items were used to assess involvement of health care providers in decision making regarding gene testing. The items included statements in which the participants were asked to characterize the “extent to which they discuss health concerns with providers,” the “extent to which their provider understand their background, needs, concerns and values,” and the “medical judgments made by the providers on their behalf” using a 4-point Likert scale (1 = strongly disagree to 4 = strongly agree). Also an item in which participants were asked if they believed their provider would recommend genetic testing if he or she believed it would cause them harm was included.

*(v) Willingness to Participate in Health-Related Research That Includes Gene Analysis*. Twenty one items were used to assess willingness to participate in health-related research that included gene analysis. Items in which participants were asked if they had ever participated in medical research were included. If participants reported that they had never participated in medical research, they were queried as about their willingness to participate and their willingness to provide personal, social, occupational and medical information, and biological specimens for genetic analysis.

*(vi) Demographic Characteristics*. Seventeen items were included within the questionnaire to elicit data reflective of the participant's gender, age, education, marital status, employment status, income, personal or family history of a chronic disease/condition, previous involvement in health-related research, insurance status, primary source of health care, and perceived health status.

Content validity and appropriateness of the questionnaire for use among the targeted population were assessed by the study investigators prior to initiation of the study.

### 3.4. Data Analysis

Descriptive and inferential statistics, computed using SPSS-PC version 20 (SPSS Inc., Chicago, IL), were used to analyze the study data. Descriptive statistics (including frequency, percentages, measures of central tendency, and measures of variability) were used to describe the characteristics of the study sample. Inferential statistics (including cross tabulations and chi-square analyses) were used to identify factors associated with willingness to participate in health-related genetic research.

### 3.5. Ethical Considerations

The research protocol of the study was reviewed and approved by the Institutional Review Board for the Protection of Human Subjects at the University of Wisconsin Milwaukee.

## 4. Results

### 4.1. Participant Profile

Of the 212 study participants, 45.8% (*n* = 97) were men and 54.2% (*n* = 115) were women (Table 1). The mean age of the study participants was 43.04 years (SD = 6.14; range 19–95). The majority of the study participants were single (62.3%, *n* = 132); employed full or part time (50.5%, *n* = 107); and had attended or completed college (60.8%, *n* = 129). Sixty four percent (*n* = 135) reported incomes of $29,999 or less and 80.7% percent (*n* = 171) reported that they were insured. When asked to describe their health status, 73.6% (*n* = 156) described their health status as “good,” “very good,” or “excellent,” and 43.4% (*n* = 92) reported a history of a chronic disease/condition.

### 4.2. Knowledge of Principles and Implications of Genetics

Ninety four percent (*n* = 192) of the African American men and women involved in the study reported that they knew little about genetics. However, data suggested that most were aware that healthy parents could bear a child with a hereditary disease; were aware of the implications of consanguinity and late parity on the expression of heritable disease/conditions and birth outcomes; and were aware of the influence of the environment on multifactorial genetic disorders (e.g., asthma, congestive heart disease, diabetes) (Table 2). In addition, when questioned about tests used for genetic analysis, most indicated that they were aware of tests used in newborn screening.

### 4.3. Beliefs Regarding the Benefits and Risks of Genetic Testing

Study participants had varied beliefs about the benefits, consequences, and risks associated with genetic testing (Table 2). Gene testing is currently used in the health care arena for carrier screening, preimplantation genetic diagnosis, prenatal diagnostic testing, newborn screening, presymptomatic testing for predicting adult-onset disorders, presymptomatic testing for estimating the risk of developing adult-onset disorders, and confirmatory diagnosis [43]. Most study participants reported that they believed that genetic testing would lead to improved treatments and improve health outcomes. Yet, several study participants expressed concerns about adverse consequences that could result from the diagnosis of a genetically-linked condition/disease (e.g., potential breech of their privacy, emotional trauma, stigma, and discrimination).

Fifty seven percent (*n* = 121) of the study participants expressed beliefs and concerns that the diagnosis of a genetically-linked condition would not remain confidential. Fifty two percent (*n* = 111) expressed beliefs and concerns about the effect of a genetically-liked condition on their family. Twenty two percent (*n* = 47) of the study participants expressed beliefs and concerns that they would not be able to emotionally handle the diagnosis of a genetically-linked condition. Thirteen percent (*n* = 28) reported that if they were found to carry a genetically-linked condition “others would view them negatively and 8.0% (*n* = 17) reported that it would cause them to feel ashamed.

### 4.4. Perceived Utility of Gene Testing

Most study participants perceived that the results of gene testing would be useful to providers attempting to make patient care decisions and to individuals attempting to improve their overall health status (Table 2). When questioned about the utility of gene testing 93.9% (*n* = 199) reported that they believed that gene testing would be useful to providers when assessing patient's health risks; 93.9% (*n* = 199) reported that they believed that gene testing would help providers in their attempts to validate (or rule out) the presence of indolent disease; and 90.1% (*n* = 191) of the study participants reported that they believed that gene testing would aid providers in their attempts to provide personalized treatments and health care. Similarly, 93.4% (*n* = 198) of the study participants reported the belief that the results of gene testing would help them make decisions about how to live a healthier life.

### 4.5. Involvement of Health Care Providers in Health Care Decisions

The merits of directive and nondirective involvement of health-care providers in decision making about gene testing have been widely reported in the literature [33, 35, 36, 44–46]. Nondirective involvement of health-care providers (relative to gene testing) implies that the patient is given relevant information about a genetic test by the health care provider and is left to make his or her own choice about testing. Directive involvement of health care providers (relative to gene testing) implies that the health-care provider reviews relevant information about a genetic test and makes the decision for the patient about testing and the patient concurs.

Most health care providers and genetic counselors when discussing gene testing, in an effort to distance gene testing from any association with “eugenics,” tend to be more inclined toward nondirective approaches. Yet, while there has been much debate about whether any discussion with patients is completely nondirective, research suggests that most clients, attempting to make decision about genetic testing and genetics related research expect information, advice and help in making decisions [35, 36].

Review of the study data revealed that the majority of the participants valued their health-care provider's knowledge and advice when making health decisions. Seventy percent (*n* = 148) of the study participants reported that they completely trusted their provider's judgment about their medical care; 89.6% (*n* = 190) reported that the medical information they received from providers was accurate and up-to-date, and 81.1% (*n* = 172) reported that they always tried to follow the provider's advice. When probed further, 67.5% (*n* = 143) reported that their providers put their medical needs above costs and all other considerations and 89.6% (*n* = 190) reported that they trusted that their provider would refer them for specialized testing if there was a need.

### 4.6. Willingness to Participate in Health-Related Research That Included Gene Analysis

Twenty percent (*n* = 43) of the study participants reported that they had been previously involved in a health-related research study. Among those who indicated that they had not been previously involved in a health-related research study, 50.3% (*n* = 169) reported a willingness to participate. Fifty one percent (*n* = 85) reported that they would be willing to complete detailed questionnaires about their family health history, social history, occupational history, psychological, and/or emotional health. Fifty three percent (*n* = 70) reported that they would be willing to allow researchers to collect environmental samples from their home. Fifty two percent (*n* = 82) reported that they would be willing to provide researchers biological specimens (e.g., tissue, blood, saliva, hair, nail clippings) for genetic analysis. Fifty four percent (*n* = 70) reported that they would be willing to participate even if the study were longitudinal and required the collection of data over several years. However 88.7% (*n* = 149) reported that they “had never been asked.”

### 4.7. Factors Associated with Willingness to Participate in Health-Related Genetics Research

Study findings implicate the impact of knowledge, perceptions, and beliefs on the willingness of African American men and women to participate in health-related genetics research (Table 3). Participants with higher levels of knowledge about genetics and heritable diseases; with understanding of the benefits, risks, and utility of genetic testing; and who had previous involvement in a health-related research study were more likely to report a willingness to engage in health-related genetic research than study participants with lesser knowledge about genetics, those with lesser knowledge, and those who had not previously been involved in a health-related research study. Participants reporting higher levels of trust in their provider's knowledge and judgment and participants reporting that their providers “listened well” to their concerns about their health and well-being were more likely to report a willingness to engage in health-related research than those who did not. In addition, as might be expected, participants reporting that they had been informed and offered the opportunity to participate in a study were more likely to express a willingness to engage in a health-related research study than those who were not.

## 5. Discussion

The use of nonprobability sampling and self-reported measures used in this exploratory study limit the generalizability of the findings. Yet, in spite of these constraints, the findings warrant careful consideration.

Increasing the involvement of minority participants in health-related research has long been identified as a national health priority. Accounts of events that have negatively impacted the engagement of African Americans have been widely reported in the scientific literature. Also are codes, guidelines, procedures, and regulations are reported to prevent these and other unethical behaviors conducted in the name of science [47, 48].

On June 10, 1993, the NIH Revitalization Act of 1993, PL 103-4 was signed into law [49]. The legislation directed the National Institutes of Health to establish guidelines for inclusion of women and minority groups and their subpopulations in NIH-funded clinical research, unless a clear and compelling rationale and justification that inclusion is inappropriate with respect to the health of the subjects or the purpose of the research. Despite the enactment of legislation specific to the inclusion of minorities in health-related research and the adoption of codes, guidelines, procedures, and regulations to prevent unethical behaviors in research and protect the rights and well-being of persons involved, the contribution of specimens to biorepositories by African Americans and the participation of African Americans in health-related genetic research are limited.

This study was designed to assess factors deemed to be essential to the engagement of African Americans in health-related research that involves gene testing. More specifically, the study was designed to assess the influence of knowledge about genetics; beliefs regarding benefits and risks of gene testing; perceptions regarding the utility of genetic testing; and the involvement of health care providers on decisions regarding participation in health-related genetic research. The results of this study support hypotheses proposed in the organizing framework relative to influence of knowledge about genetics; beliefs regarding benefits and risks of gene testing; perceptions regarding the utility of genetic testing; and the involvement of health care providers on decisions relative to participation in health-related genetics research and the willingness to engage in health-related genetic research. As hypothesized, study findings revealed that knowledge about genetic research, perceptions regarding the utility of genetic testing, beliefs regarding the benefits and risks of genetic testing, and previous involvement in research are associated with participant's willingness to engage in health-related genetic research. Study participants reporting higher levels of trust and engagement with providers were more likely to report a willingness to engage in health related research than those who did not. The most surprising were the findings that the vast majority of the African American men and women involved in the study reported support of health-related genetic research; however, most indicated that they had “never been asked.”

This study is unique in that it attempted to highlight factors associated with the participation of African Americans in health-related research as well the processes, outcomes, and principles of informed decision making. The findings suggest the need for research that further examines factors presumed to be associated with the involvement of African Americans in health-related genetic research. The most important appears to be the need for research that explores the manner and extent to which African Americans are informed about the benefits, risks, and utility of genetic testing; research that explores the manner and extent to which African Americans are informed and afforded the opportunity to participate in health-related genetic research; and research that explores the manner and extent to which the decisions of African American men and women relative to participation in health-related genetic research (as assured during the consenting process) are supported. The findings also suggest that the need for strategies to better inform African Americans of opportunities to contribute health information and biological specimens to biorepositories and to better engage researchers and health care providers in efforts to inform, recruit, and support African American men and women willing to participate in health-related genetic research. Without the design of interventions to better inform, recruit, engage, and support the decisions of African Americans (and persons representing other minority population groups) willing to participate in health-related genetic research it would be reasonable to anticipate that current trends relative to their involvement will remain unchanged.

## Figures and Tables

**Table 1:** Profile of the study participants (*N* = 212).

Background characteristics	*n *	(%)	
Age in years			Mean = 43.04; SD = 6.14
19–29	38	17.9	
30–39	47	22.2	
40–49	61	28.8	
50≥	66	31.1	
Gender			
Male	97	45.8	
Female	115	54.2	
Marital status			
Married	34	20.2	
Partnered	9	4.2	
Single	133	63.5	
Widowed	9	4.2	
Divorced/separated	24	11.3	
Education			
High school or less	83	39.2	
Some college	84	39.6	
College graduate	31	14.6	
Graduate degree	14	6.6	
Employment			
Full time	80	37.7	
Part time	27	15.1	
Unemployed	59	30.2	
Disabled, not able to work	36	17.0	
Income			Median: $20,000–$29,999
<$5,000–$9,999	60	28.3	
$10,000–$29,999	75	35.4	
$30,000–$49,999	36	17.0	
$50,000–$69,999	21	5.7	
$70,000+	5	2.4	
Insurance status			
Insured	171	80.7	
Uninsured	36	17.0	
Perceived health status			
Excellent	29	13.7	
Very good	55	25.9	
Good	81	38.2	
Fair	35	16.5	
Poor	12	5.7	
History of chronic disease			
Yes	92	43.4	
No	115	54.2	
Regular healthcare provider		
Yes	165	77.8	
No	46	21.7	

**Table 2:** Knowledge, perceptions, beliefs, and willingness to engage in health-related genetic research (*N* = 212).

Variable	*n *	(%)	
*Knowledge about principles and implications of genetics *			Range = 3–11; mean = 8.49; SD = 1.63
Knowledge of implications of consanguinity on heritable diseases/conditions	197	92.9	
Knowledge of implications of late parity on heritable diseases/conditions	167	78.8	
Knowledge of influence of the environment on genetic disorders	186	87.7	
Knowledge of tests and procedures used in genetic testing			
Breast tumor analysis for BRCA1/BRCA2 mutations	61	28.8	
Amniotic fluid analysis for fetal defects	174	82.1	
Capillary blood analysis of newborns genetic diseases in newborns	144	67.9	

*Beliefs about advantages of genetic testing *			Range = 0–6; mean = 3.4; SD = 2.2
Believe that testing would provide important information	112	52.8	
Believe that testing would lead to improved risk assessment	170	80.2	
Believe that testing would lead to improved treatment	140	66.0	

*Beliefs about disadvantages of genetic testing *			Range = 13–53; mean = 34.3; SD = 7.14
Believe that testing would breach privacy and confidentiality	121	57.1	
Believe that abnormality would have negative impact on the family	111	52.3	
Believe that testing would cause emotional trauma	47	22.2	
Believe that results would cause other to view them negatively	28	13.2	
Believe that results would result in feelings of being “singled out”	19	9.0	
Believe that genetic abnormality would cause a sense of shame	17	8.0	

*Perceived utility of gene testing *			Range = 0–6; mean = 5.6; SD = 1.1
Believe that gene testing would provide information about disease risk	199	93.9	
Believe that gene testing would personalize screening/diagnostics	199	93.9	
Believe that gene testing would lead to personalize treatments	191	90.1	
Believe that gene testing would provide health information for lifestyle intervention	198	93.4	

*Provider trust and involvement in health care decisions *			Range = 31–80; mean = 60.5; SD = 9.1
Trust the provider's medical judgment	148	69.8	
Trust that providers place their needs above other considerations	143	67.5	
Providers refer for specialized testing as needed	190	89.6	
Follow provider's medical advice	172	81.1	

*Willing to participate in health-related study that included gene analysis *	116	54.7	
Willing to complete detailed questionnaires about family history	113	55.3	
Willing to allow the collection of environmental samples	94	44.3	
Willing to provide biological specimens for gene analysis	109	51.4	
Willing to participate in longitudinal studies	94	44.3	

**Table 3:** Factors associated with willingness to engage in health-related genetic research (*N* = 212).

Variable	*χ* ^2^	df	*P *
Knowledge about principles and implications of genetics			
Awareness of implications of late parity on heritable diseases	7.346	2	.025
Understanding of patterns of transmission of heritable disorders	4.07	1	.045
Awareness of tests and procedures used in genetic testing	4.343	1	.037
Understanding of benefits and risks of genetic testing	49.787	35	.050
Perceptions regarding utility of genetic testing	4.176	1	.042
Provider trust and involvement in health care decisions	8.648	3	.034
Previous involvement in health-related research	4.226	1	.040
